# Investigating and evaluating evidence of the behavioural determinants of adherence to social distancing measures – A protocol for a scoping review of COVID-19 research

**DOI:** 10.12688/hrbopenres.13099.2

**Published:** 2020-09-10

**Authors:** Chris Noone, Nikolett Warner, Molly Byrne, Hannah Durand, Kim L. Lavoie, Brian E. McGuire, Jenny Mc Sharry, Oonagh Meade, Eimear Morrissey, Gerry Molloy, Laura O'Connor, Elaine Toomey

**Affiliations:** 1 School of Psychology, National University of Ireland, Galway, Galway, Galway, H91 TK33, Ireland; 2 Department of Psychology, University of Quebec at Montreal, Montreal, Quebec, H2L 2C4, Canada; 3 Montreal Behavioral Medicine Centre, CIUSSS-NIM – Hôpital du Sacre-Coeur de Montreal, Montreal, Quebec, H4J 1C5, Canada; 4 School of Medicine, National University of Ireland, Galway, Galway, Galway, H91 TK33, Ireland; 5 School of Allied Health, University of Limerick, Limerick, Limerick, V94 T9PX, Ireland

**Keywords:** COVID-19, coronavirus, social distancing, physical distancing, pandemic

## Abstract

**Background:** The WHO has declared the outbreak of coronavirus disease 2019 (COVID-19) as a pandemic. With no vaccine currently available, using behavioural measures to reduce the spread of the virus within the population is an important tool in mitigating the effects of this pandemic. As such, social distancing measures are being implemented globally and have proven an effective tool in slowing the large-scale spread of the virus.

**Aim:** This scoping review will focus on answering key questions about the state of the evidence on the behavioural determinants of adherence to social distancing measures in research on COVID-19.

**Methods:** A scoping review will be conducted in accordance with guidelines for best practice. Literature searches will be conducted using online databases and grey literature sources. Databases will include Medline, Web of Science, Embase and PsycInfo, alongside relevant pre-print servers. Grey literature will be searched on Google Scholar. Screening, data extraction and quality appraisal will be conducted by members of the research team, with any discrepancies resolved by consensus discussion. Quality appraisal will be conducted using the Cochrane’s ROBINS-I tool, the Cochrane Risk of Bias tool, and the JBI Critical Appraisal Checklist where appropriate. Results will be analysed by mapping findings onto the Theoretical Domains Framework and visualising characteristics of the included studies using EviAtlas. This scoping review is pre-registered with Open Science Framework.

**Conclusions** The results of this study may facilitate the systematic development of behavioural interventions to increase adherence to social distancing measures.

## Introduction

Coronavirus disease 2019 (COVID-19) has had a devastating effect globally since it was first identified in China in December 2019 ( Johns Hopkins University, 2020). While several vaccines against SARS-COV-2, the virus which causes COVID-19, are in development, there are none currently available ( WHO, 2020). The lack of a vaccine means that behavioural strategies for reducing the transmission of COVID-19 are vital to the global pandemic response ( Michie *et al.*, 2020). Some refer to these strategies collectively as a “behavioural vaccine” ( Speight *et al.*, 2020).

Societal and community-level strategies for controlling the pandemic including various levels of lockdown and quarantine focus on preventing physical contact between people through public health recommendations, environmental restructuring or legal mandates ( Perkins & Espana, 2020). These and other activities that prevent or reduce the frequency and closeness of contact between people as a means of interrupting disease transmission are often collectively referred to as social distancing measures ( Kinlaw & Levine, 2007). Individual-level preventative strategies include effective handwashing, properly disinfecting surfaces, coughing and sneezing into a tissue, wearing protective masks, avoiding touching one’s face and keeping a physical distance from others – which is *also* often referred to as social distancing, though many now refer to this behaviour as physical distancing ( West *et al.*, 2020).

While understanding and developing interventions for handwashing, mask-wearing and cough and sneeze etiquette have been the focus of research in psychology previously and in relation to other infectious diseases, less is known about how to effectively encourage behaviours related to social distancing and research in the context of COVID-19 is obviously just emerging ( Berry & Fournier, 2014; Jefferson *et al.*, 2007; Lunn *et al.*, 2020; Luong Thanh *et al.*, 2016). Adherence to measures which increase social distance is vital to the success of exit strategies of countries that underwent lockdown and efforts to end the COVID-19 pandemic ( Gilbert *et al.*, 2020). Social distancing measures have been shown to reduce the spread of COVID-19 ( Courtemanche *et al.*, 2020). Recent evidence syntheses have focused on the efficacy of social distancing measures ( Chu *et al.*, 2020; Mahtani *et al.*, 2020; Regmi & Lwin, 2020). One found observational support for keeping a distance of at least 1m, with the chances of COVID-19 transmission decreasing with distance ( Chu *et al.*, 2020). Another found support from systematic reviews for self-isolation of ill and exposed individuals, and strategies for reducing people’s social contacts and mobility ( Mahtani *et al.*, 2020). Our scoping review will focus on the determinants that potentially increase adherence to social distancing measures, which may inform the development of behavioural interventions to increase adherence to these measures, and thereby increase their effectiveness. Understanding how people successfully adhere to these measures will also be vital to the control of future pandemics.

Behavioural interventions aimed at ensuring high levels of adherence to social distancing guidelines (and other preventative behaviours) have been described as “urgently needed” ( Glasziou *et al.*, 2020). However, as of April 2020, only a handful of studies had been registered to test behavioural interventions for preventing COVID-19 transmission – and none focused on increasing adherence to social distancing measures ( Hoffmann & Glasziou, 2020). It is crucial that behavioural interventions that can reduce the transmission of COVID-19 are rapidly developed, tested, optimised and implemented in a systematic and evidence-based manner.

A vital step in developing behavioural interventions, regardless of the development framework being employed, is collating the relevant evidence regarding the potential determinants of the behaviour that needs to be changed ( O’Cathain *et al.*, 2019a). It is therefore crucial to facilitate the development and testing of such interventions by mapping and evaluating the research on the behavioural determinants of adherence to social distancing measures. The Theoretical Domains Framework is a useful tool for mapping the determinants of behaviours and linking them to specific intervention functions ( Michie *et al.*, 2005). It summarises 128 constructs derived from 33 theories of health behaviour into 14 domains. Thus, it provides a method for collating and summarising research on determinants of health behaviours such as adherence to social distancing measures.

Emerging areas of research are often described using scoping review methods as they allow for a broader focus than systematic reviews and present results in descriptive formats that highlight what kinds of evidence exist, where there are evidence gaps, and the quality of the existing evidence ( Nyanchoka *et al.*, 2019). Scoping reviews are also specifically indicated when there is a need to clarify the key constructs and operational definitions employed in an area of research, to examine the ways in which research in an emerging area is being conducted and to identify the factors associated with a specific concept ( Munn *et al.*, 2018).

The COVID-19 pandemic has caused an exponential increase in research on ways of tackling this crisis and concerns have been raised about the level of research waste that this has produced ( Glasziou *et al.*, 2020). Given that there are also concerns about the readiness of psychology as a discipline to contribute to policymaking in emergencies ( IJzerman *et al.*, 2020), it is imperative that we consider this growth in research carefully and evaluate the quality of its products – particularly in new areas such as social distancing in research on COVID-19. This scoping review will focus on answering key questions about the state of the evidence on the behavioural determinants of adherence to social distancing measures in research on COVID-19.

## Protocol

This scoping review will be carried out in accordance with guidance from the Joanna Briggs Institute, which builds on previous guidance on best practice in scoping review methodology ( Arksey & O’Malley, 2005; Levac *et al.*, 2010; Peters *et al.*, 2019) and reported in accordance with PRISMA-ScR guidance ( Tricco *et al.*, 2018). This protocol is structured according to the steps suggested by Arksey & O’Malley (2005). Any deviations from this protocol will be tracked on the review’s Open Science Framework project; the protocol is pre-registered at https://doi.org/10.17605/OSF.IO/TMKUX.

### Stage 1: Identifying the research question

We aim to address the following research questions (RQ) relating to social distancing in research on COVID-19:

RQ1. In what ways have social distancing measures been defined and how has adherence to these measures been operationalised in research on their behavioural determinants conducted in research on COVID-19?
RQ2. What behavioural determinants of adherence to social distancing measures have been studied in research on COVID-19?
RQ3. How do the behavioural determinants of adherence to social distancing measures that have been studied in research on COVID-19 map onto the Theoretical Domains Framework (TDF; Cane *et al.*, 2012)?
RQ4. What is the quality of the evidence from the included studies in this scoping review?
RQ5. What study designs have been used to study the behavioural determinants of adherence to social distancing measures in research on COVID-19?
RQ6. Where has this research taken place?
RQ7. What gaps exist in the literature that need to be addressed in future research on social distancing measures?

### Stage 2: Identifying relevant studies

***Eligibility criteria***. Studies must focus on human participants, but no further exclusions on the basis of participant characteristics will be made. Included studies must measure adherence to social distancing measures (i.e. quarantine, lockdown, and physical distancing) and include potential behavioural determinants of adherence to these measures as independent variables (i.e. as either an observed or manipulated variable). By behavioural determinants, we mean any intrapersonal, interpersonal, community, societal or cultural influences on behaviour as commonly represented by ecological models of health ( Golden & Earp, 2012). Included studies must have collected primary data using quantitative designs. The included studies must have specifically been conducted in relation to COVID-19 (see Table 1). There will be no restriction on languages. We will use Google Translate to aid in the screening and data extraction of sources that are not reported in English as there is evidence that this is an effective approach ( Jackson *et al.*, 2019). A definitions and elaboration document has been developed based on these criteria to aid screening (see *Extended data* ( Noone *et al.*, 2020)).

**Table 1.** Inclusion criteria for this study.

Inclusion criteria	Details
Participants	Any human participant
Concept	Potential behavioural determinants of adherence to social distancing measures
Context	The outbreak of COVID-19
Sources	Any study that employs a quantitative design and collects primary data

***Information sources***. We will identify potentially relevant published literature by searching Medline, PsycInfo, Embase, and Web of Science Core Collection, as this combination of databases has been recommended for adequate and efficient search coverage ( Bramer *et al.*, 2017). We will also identify potentially relevant pre-prints by searching PsyArXiv, medRxiv, SocArxiv and Preprints.org. We will search ClinicalTrials.gov and the WHO International Clinical Trials Registry Platform to identify any potentially relevant trials. Grey literature will be searched for using Google Scholar, according to the guidance from Haddaway & colleagues (2015), which suggests using the title-only search option and screening the first 1000 records.

***Search strategy***. The Medline search strategy for the review was developed with assistance from a research support librarian and includes terms related to COVID-19 and social distancing measures. The searches will be restricted to 2020 to ensure that only sources relevant to the COVID-19 pandemic are identified. The Peer Review of Electronic Search Strategies checklist ( McGowan *et al.*, 2016) was applied to this strategy by an independent information specialist. Once the suggested adjustments were applied, the search strategy was translated to the other databases using the Polyglot Search Translator ( Clark *et al.*, 2020). Separate search strategies were developed for each pre-print server and Google Scholar. We will use the medrxivr app to search medRxiv ( McGuinness, 2020). For each of the other sources, we will use the native search interface. The full search strategy is documented in the Extended data ( Noone *et al.*, 2020).

***Reference management***. Search results from Medline, PsycInfo, Embase, and Web of Science will be exported to .ris files and then imported to Zotero. Search results from both trial registries, each pre-print server and Google Scholar will be imported to Zotero using the Zotero Connector. Specific folders for each literature source will be created in the Zotero Group library for this project, which is available at https://bit.ly/BDSDA_Library. All search results will be exported to a single .ris file so that deduplication can be conducted using the DeDuplicator tool within the Systematic Review Accelerator suite ( Rathbone *et al.*, 2015). The deduplicated library will then be exported for screening.

### Stage 3: Study selection

Screening will be conducted within Covidence ( Covidence, 2019). The screening process will be piloted initially – 25 titles and abstracts will be selected at random and the entire research team will screen these using the predefined eligibility criteria and definitions/elaboration document. If any discrepancies are identified, these will be discussed within the team and modifications will be made to the eligibility criteria and the definitions and elaboration document. Screening will then begin once an agreement rate of 75% or greater is reached based on the screening of a further 25 titles and abstracts. The screening process will involve two reviewers screening each title and abstract, with conflicts resolved by consensus or a third reviewer. Full texts will also be screened in duplicate with conflicts resolved by consensus or a third reviewer.

### Stage 4: Charting the data

The research team will design a data charting tool, as set out by the PRISMA-ScR Checklist ( Tricco *et al.*, 2018), to which the following information will be extracted by two members of the research team:

- Author(s)
- Year of publication
- Country of origin (i.e. where the study was conducted)
- Funding
- Aims/purpose
- Time of data collection
- Context (e.g., description of local COVID-19 impact at time of data collection, description of relevant public health policies in place; if available in the study report)
- Population
- Sample size
- Study design
- Pre-registration (if any)
- Specific theory (if any)
- Intervention type, comparator and details of these (e.g., duration of the intervention; if applicable).
- Outcomes and details of these (e.g., how measured)
- Key findings that relate to the scoping review question/s.

The data charting tool will be independently piloted by two reviewers who will conduct full data extraction on five sources chosen to cover the diversity of different study types included.

Any discrepancies that arise will be discussed by the full team before proceeding with the data extraction process. As per the iterative nature of scoping reviews, it is expected that this tool may be adjusted during this process to ensure accurate representation of all data sources. Data from each included source will be extracted by one reviewer and checked by another. All data extracted will be compiled into a summary spreadsheet.

***Quality assessment***. We will use the following quality appraisal tools for studies included in the review: the Cochrane Risk of Bias Tool ( Sterne *et al.*, 2019) for randomised controlled trials, the Cochrane ROBINS-I Tool ( Sterne *et al.*, 2016) for quasi-experimental studies, and the Joanna Briggs Institute Critical Appraisal Checklist ( Moola *et al.*, 2020) for analytical cross-sectional research.

### Stage 5: Collating, summarising, and reporting of results

To visually represent study selection and reasons for exclusion at full text review, a PRISMA flow diagram will be presented. The diversity of definitions of social distancing measures and the operationalisations of adherence to these measures will be recorded in a table (RQ1). A table of study characteristics will summarise the aim, design and results (if available) of each study (RQ2). A framework analysis using the TDF ( Cane *et al.*, 2012) will be used to summarise and report the types of evidence available regarding the behavioural determinants of adherence to social distancing measures that have been studied in research on COVID-19. Two reviewers will independently judge which domains of the TDF are most applicable to each behavioural determinant of adherence to social distancing measures reported in the included studies. Additional headings will be employed should the framework not be sufficient in representing the data (RQ3). While there is little consensus on the exact definition of what constitutes an evidence gap map ( Miake-Lye *et al.*, 2016), for the purpose of this review, we aim to produce a visual depiction of current research and any gaps in the literature, alongside an assessment of study quality (RQ4). This will be presented using a bubble plot, whereby the colour of and size of each bubble will represent research type and research quality. A heat map will present counts of the different research designs used in the included studies (RQ5). A geographical map will be produced to visualise the volume of included studies carried out in different countries (RQ6). Knowledge gaps will be represented through the development of an evidence gap map (RQ7). We will also produce a timeline of the studies based on the reported time of data collection. The visualisations described above will be produced using EviAtlas, an open science tool for mapping and graphing study characteristics ( Haddaway *et al.*, 2019). Clusters and heat maps of frequently occurring terms in the included studies will be visualised using VOSviewer ( van Eck & Waltman, 2010). Strengths and limitations to the review will be discussed, alongside future recommendations for research.

### Step 6: Consultation with stakeholders

We consider the primary stakeholders in this study to be the researchers developing work in this area, in particular those who develop behavioural interventions. We will invite open consultation and seek comments on this article. We will disseminate this invitation through relevant professional societies (e.g. the International Society for Behavioural Medicine, the European Health Psychology Society, the Asian Congress of Health Psychology), social media networks and mailing lists. We will summarise all feedback received and record any changes in the study that are made as a result.

### Dissemination

To facilitate rapid dissemination, the results of this review will be reported in a pre-print which will be uploaded to PsyArXiv. This report will also be submitted to a peer-reviewed journal.

## Discussion

Developing interventions that effectively increase adherence to social distancing measures is vital to the success of efforts to tackle the COVID-19 pandemic and will contribute to preparedness for future pandemics. This scoping review will systematically collate and describe the available evidence regarding the behavioural determinants of adherence to social distancing measures. It will also highlight gaps in this area of research. This may reduce research waste by making it easier to avoid the unnecessary duplication of work and instead contribute to cumulative research on this topic.

Ideally, the results of this study will facilitate the systematic development of behavioural interventions to increase adherence to social distancing measures based on behavioural evidence and theory using established approaches such as Intervention Mapping ( Bartholomew Eldredge, 2016) and the Behaviour Change Wheel ( O’Cathain *et al.*, 2019b; Michie *et al.*, 2014). These interventions should then be tested within methodological frameworks that can rapidly and efficiently identify the optimal set of components that the interventions should contain. The Multiphase Optimisation Strategy ( Collins *et al.*, 2011) and the Agile Science process ( Hekler *et al.*, 2016) are two such frameworks that make intelligent use of innovative research designs such as fractional factorial experiments, microrandomised trials and sequential multiple assignment randomised trials.

To facilitate this work, this review will produce an accessible summary of how social distancing measures are defined and how adherence to these measures has been operationalised in research conducted on COVID-19. It will also identify the behavioural determinants that have been studied in relation to adherence to social distancing measures and map them to the TDF. Finally, it will analyse and visualise key characteristics of the included studies.

## Data availability

### Underlying data

No underlying data are associated with this article.

### Extended data

Open Science Framework: Investigating and evaluating evidence of the behavioural determinants of adherence to social distancing measures – A scoping review of COVID-19 research. https://doi.org/10.17605/OSF.IO/TMKUX ( Noone *et al.*, 2020).

This project contains the following extended data:

- Cochrane ROB Tool.pdf (Risk of bias tool to be used).
- Codebook.docx
- Data Extraction Form.xlsx (Blank data extraction form).
- Eligibility Criteria.docx
- EMBASE.docx (Search strategy for EMBASE).
- JBI Critical Appraisal.pdf (JBI Critical Appraisal check
- MEDLINE.docx (Search strategy for MEDLINE).
- Pre-print, Grey Lit and Registry Search Strategies.docx (Search strategies for pre-prints, grey literature and registries).
- PRESS 2015 checklist for search strategies.docx
- PSYCINFO.docx (Search strategy for PSYCINFO).
- ROBINS-I Tool.pdf (Blank ROBINS-I assessment tool).
- Web of Science.docx (Search strategy for Web of Science).

Extended data are available under the terms of the Creative Commons Attribution 4.0 International license (CC-BY 4.0).
