# Supplementary material for: Investigating and evaluating evidence of the behavioural determinants of adherence to social distancing measures – A protocol for a scoping review of COVID-19 research
Source: HRB Open Res. 2020 Sep 10;3:46. Originally published 2020 Jul 21. [Version 2] doi: 10.12688/hrbopenres.13099.2 (PMC7406949; doi:10.12688/hrbopenres.13099.2)
Supplement: Peer review history

# Peer review: Reviewer response for version 1

**DOI:** 10.21956/hrbopenres.14201.r27703

**Published:** 2020-08-05

**Authors:** Alexandra L. Dima, Luiza Siqueira do Prado

**Affiliations:** 1 Health Services and Performance Research EA 7425, Université Claude Bernard Lyon 1, Lyon, France

This is indeed a very well written protocol of a scoping review on determinants of adherence to social distancing measures during the COVID-19 pandemic. It provides a complete description of review items and fully adheres to the principles and practices of open science. They provide access to search strategies and the records retrieved, which, along with the codebook and the data extraction form, effectively ensure reproducibility. While we acknowledge that it is difficult to find points of improvement to such a thorough work, we would like to suggest the following points for authors’ consideration:

1. One of the study eligibility criteria is that determinants should be included as independent variables (IV). We understand it as "excluding studies where determinants are dependent variables”, but it may be interpreted also as selecting only quantitative observational studies. In the description of Stage 4, we see that experimental/interventional designs are included. In these studies, the intervention (vs control) would be the IV in the statistical analysis and determinants might be explicit or implicit in the choice of intervention components, or secondary outcomes. For some determinants, such as ‘intention to distance physically’, it might be difficult to tell whether the included studies consider it a determinant or a proxy for behaviour. Moreover, interventions to change determinants (with a view to changing behaviour) might also be informative. An explanation or further elaboration of this criterion might be useful.
2. The point above has implications for the data extraction – as it reads now in the codebook, determinants are only extracted for observational studies. But they could be implicit in intervention studies or explicitly stated in a logic model of the intervention. We agree with the first reviewer that it would be useful in this case to code the change techniques/methods used – and how they were linked with determinants by the authors of the original study, if at all.
3. The focus of this review is on quantitative research, but in this emerging crisis qualitative inquiry is equally valuable for identifying determinants (e.g. Williams *et al.* ^1^). It would be useful to state why qualitative studies are excluded, or perhaps include them as well? If the authors prefer to focus only on quantitative information, mentioning this in the abstract will be useful for readers.
4. The term used for determinants is “behavioral determinants”. Are there ‘non-behavioral determinants’ in this context which would be excluded? The term “behavioral determinants” is used in public health to denote the impact of behaviour on health outcomes (as opposed to social, environmental, etc.), but in the context of studying determinants of a behaviour (distancing) can we talk about non-behavioral or is it just intended as ‘all determinants of this behaviour’? Would for example socio-demographic characteristics be excluded if they appear as IVs in a regression model with distancing as DV? Perhaps other distinctions to consider in the review would be between modifiable-non modifiable, and individual-environmental (and in the environment there could be different actors and behaviours facilitating social distancing). These would inform intervention by recommending modifiable determinants and identifying which actors it could target.
5. Following on the previous point, presence of interactions between determinants and background characteristics could be useful to code to guide tailoring of interventions.
6. In this topic, grey literature is likely to be very important. The Appendix with grey literature searches includes only ‘distancing’ as search term, while for the other sources it has already been expanded to other keywords (isolation, quarantine, etc.). It would be useful to use these other terms here as well. Moreover, from own experience with Google Scholar, title only searches might miss important contributions. It would perhaps be useful to compare with ‘allintext’ for relevance of entries.
7. The abbreviations RQ1, RQ2, etc., in "Stage 5: Collating, summarising, and reporting of results" refer to the research questions presented in Stage 1. Please add abbreviation where it first appears in the text. 

Is the study design appropriate for the research question?

Yes

Is the rationale for, and objectives of, the study clearly described?

Yes

Are sufficient details of the methods provided to allow replication by others?

Yes

Are the datasets clearly presented in a useable and accessible format?

Yes

Reviewer Expertise:

Behaviour change, health psychology, public health, health services research.

We confirm that we have read this submission and believe that we have an appropriate level of expertise to confirm that it is of an acceptable scientific standard.

## References

[1] : Public perceptions and experiences of social distancing and social isolation during the COVID-19 pandemic: a UK-based focus group study. *BMJ Open*.2020;10(7) : 10.1136/bmjopen-2020-039334 10.1136/bmjopen-2020-039334 PMC738731032690752

# Response

**Published:** 2020-08-28

**Authors:** Chris Noone

**Affiliations:** NUI Galway, Ireland

We thank the reviewers for their constructive review of our protocol. In the revised version of the protocol, we have clarified that we think of behavioural determinants as the full range of factors that can be conceptualised within ecological models of health behaviour as potentially influencing behaviour. We also clarified that the determinants may be present in studies as either observed or manipulated variables. While we agree that categorising the potential determinants found as modifiable or non-modifiable, we feel that in the absence of behavioural intervention evidence, it is impossible to judge which potential behavioural determinants of adherence to social distancing measures can be modified. We have revised our codebook and data extraction form and these are now available on our  OSF project page. Unfortunately, there are not enough members of our team with BCT coding experience available to carry this out. This would be an interesting follow-up study and we will note this in the discussion of our results paper and our data will be available for other teams who may wish to do this work. While we agree that qualitative research is vital to understand adherence to social distancing measures, we considered that it would be more feasible to focus specifically on quantitative research. We acknowledge that our grey literature search was limited by not including all of the terms related to social distancing and we will broaden the search if there are updates of this scoping review. Finally, we have added the abbreviation of research question at it’s first mention.

# Peer review: Reviewer response for version 1

**DOI:** 10.21956/hrbopenres.14201.r27701

**Published:** 2020-07-24

**Authors:** Emma Norris

**Affiliations:** 1 Department of Clinical Sciences, Brunel University, Uxbridge, UK

This paper presents a thorough, clear protocol of a scoping review exploring the behavioural determinants of adherence to social distancing measures in research conducted during COVID-19. The authors are making clear attempts to make the results of this review as accessible as possible, using new visualisation techniques for evidence synthesis (EviAtlas), pre-registering their work, searches and data collection tools, as well as planning to pre-print the results to enable faster access by policy-makers etc.

 My comments are:

1. There have been various other attempts to synthesise what is known about social distancing in COVID-19. Although these clearly do not focus on the behavioural determinants of social distancing, other related reviews should be mentioned and your own review distinguished from them e.g Chu *et al*. 2020 ^1^;  Mahtani *et al*. 2020 ^2^ ; Regmi & Lwin 2020 ^3^.
2. As well as coding for TDF, could you also code for the Behaviour Change Techniques used in studies using the BCTTv1? This would be particularly insightful for intervention studies.
3. Will inter-rater reliability of double-coding be performed, such as using Krippendorrf’s alpha? Such reliability calculations are typical for double-coding in systematic reviews and for behaviour change techniques e.g Howlett *et al*. 2018 ^4^.
4. PRISMA-ScR Checklist is described to be used for charting data – will this also be used as a reporting checklist for the final write-up?
5. Typo: ‘must be measure’ should be ‘must measure’.

Is the study design appropriate for the research question?

Yes

Is the rationale for, and objectives of, the study clearly described?

Yes

Are sufficient details of the methods provided to allow replication by others?

Yes

Are the datasets clearly presented in a useable and accessible format?

Yes

Reviewer Expertise:

Behaviour change, health psychology, evidence synthesis, open science.

I confirm that I have read this submission and believe that I have an appropriate level of expertise to confirm that it is of an acceptable scientific standard.

## References

[1] : Physical distancing, face masks, and eye protection to prevent person-to-person transmission of SARS-CoV-2 and COVID-19: a systematic review and meta-analysis. *The Lancet*.2020;395(10242) : 10.1016/S0140-6736(20)31142-9 1973-1987 10.1016/S0140-6736(20)31142-9 PMC726381432497510

[2] : What is the evidence for social distancing during global pandemics? A rapid summary of current knowledge. *Oxford COVID-19 Evidence Service*.2020;

[3] : Impact of social distancing measures for preventing coronavirus disease 2019 [COVID-19]: A systematic review and meta-analysis protocol. *medRxiv*.2020; 10.1101/2020.06.13.20130294 10.1101/2020.06.13.20130294

[4] : Are physical activity interventions for healthy inactive adults effective in promoting behavior change and maintenance, and which behavior change techniques are effective? A systematic review and meta-analysis. *Translational Behavioral Medicine*.2019;9(1) : 10.1093/tbm/iby010 147-157 10.1093/tbm/iby010 PMC630556229506209

# Response

**Published:** 2020-08-28

**Authors:** Chris Noone

**Affiliations:** NUI Galway, Ireland

We’re very grateful for these constructive comments. We have revised the protocol to highlight other recent evidence syntheses focused on social distancing measures and distinguished our scoping review from these. We appreciate the suggestion of including BCT coding of any interventions found. Unfortunately, there are not enough members of our team with BCT coding experience available to carry this out. This would be an interesting follow-up study and we will note this in the discussion of our results paper and our data will be available for other teams who may wish to do this work. Our limited resources are also forcing us to switch from data extraction in duplicate to the work of one reviewer being checked by another, so inter-rater reliability is no longer relevant. Regarding the use of the PRISMA-ScR for reporting, this is already mentioned in the first paragraph of the protocol section. Finally, thanks for pointing out the typo to us. It is now correct.
